# Debilitating Metastatic Spindle Cell Carcinoma of the Breast

**DOI:** 10.7759/cureus.3864

**Published:** 2019-01-10

**Authors:** Jonathan S Alexander, Hamza Hashmi, Samuel B Reynolds

**Affiliations:** 1 Internal Medicine, University of Louisville School of Medicine, Louisville, USA; 2 Oncology, James Graham Brown Cancer Center, University of Louisville School of Medicine, Louisville, USA

**Keywords:** spindle, cell, carcinoma, breast, metastatic, screening, early, detection

## Abstract

Spindle cell carcinoma of the breast is a rare malignancy. If diagnosed and treated in a timely manner, it is generally associated with a good prognosis. Herein, we have presented an interesting case of metastatic spindle cell carcinoma of breast origin, with extensive metastasis and an unusually aggressive disease course. We also discussed refractory hypoglycemia as a fatal complication of highly metabolically active malignancy. Lastly, we briefly explored the importance of seeking medical attention for early detection and treatment and the need to address psychosocial barriers that influence breast cancer screening.

## Introduction

Spindle cell carcinoma is a rare breast tumor, with an estimated yearly incidence <0.5%. It is especially uncommon in patients with no prior history of radiation therapy to the breast. It tends to have a favorable prognosis, with lymph node involvement relatively uncommon, provided it is detected early and treated appropriately [1]. Herein, we have presented a rare case of metastatic spindle cell carcinoma of breast origin in a patient with a particularly aggressive disease course and extensive metastases.

## Case presentation

**Figure 1:**
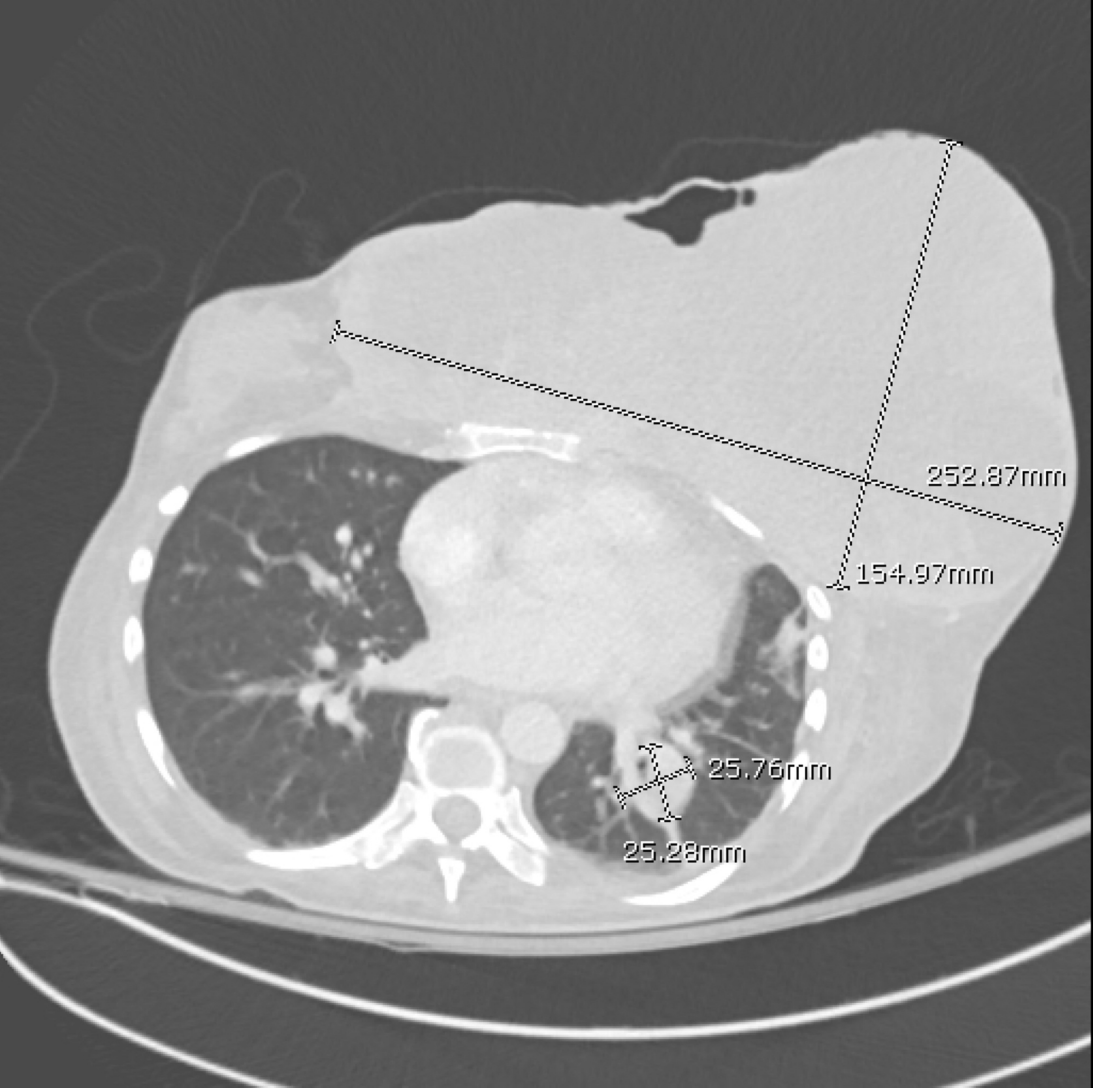
CT chest axial view showing extensive primary tumor burden as well as lung metastasis CT: computed tomography

A 50-year-old woman with no significant prior medical history presented with a six-month history of a rapidly enlarging left-sided breast mass that, in recent weeks, had become increasingly painful and ulcerative in nature. She had initially felt a small lump in her breast but chose not to have it evaluated by a physician until the pain from the mass became intolerable. On presentation, she was tachycardic and mildly tachypneic with a low-grade fever. The breast mass had evidence of extensive necrosis with active drainage of blood and pus. The patient was unable to ambulate due to the weight of the tumor and the accompanying back pain. Laboratory testing revealed lactic acid levels of 13 mmol/L (normal <1.70) and a white cell count of 21.9 K/uL (normal range: 4.1-10.8). Computerized tomography (CT) imaging showed extensive primary tumor burden that crossed the midline and measured 25.3 x 21.2 x 15.5 cm, as well as an additional smaller tumor in the right breast (Figure 1). Pulmonary, osseous, and nodal metastases were also noted. Tissue biopsy revealed triple negative, poorly differentiated, spindle cell carcinoma with immunochemistry positive for cytokeratin (CK) AE1/3, CK 5/6, CK 14, and tumor protein p63. Treatment was initially focused on supportive care that included broad-spectrum antibiotics to cover any soft tissue infection that may have been contributing to the patient’s metabolic derangement. Chemotherapeutic and surgical interventions were deemed futile due to the advanced stage of the disease. It was determined that radiation therapy could be of palliative benefit and thus, the patient underwent appropriate simulation and a single treatment to the affected site, with additional treatments planned for future. The patient’s hospital course was further complicated by persistent hypoglycemia that began on day three after admission. Despite being maintained on a continuous infusion of 5% dextrose in normal saline, the patient’s blood glucose would recurrently drop as low as 42 mg/dl (normal range: 70-110), requiring intermittent oral glucose administration and a change in intravenous fluids to 10% dextrose in water. An extensive endocrine workup, which included checking serum levels of insulin, proinsulin, insulin-like growth factor 1, c-peptide, adrenocorticotropic hormone, and cortisol, revealed no significant abnormalities. The patient continued to decline with a worsening of her metabolic acidosis and an increasing need for respiratory support. After a detailed meeting, the family decided to pursue comfort measures. The patient passed away 11 days after initial presentation.

## Discussion

Being a rare disease, most details surrounding spindle cell carcinoma of the breast are the result of published case reports and series. There is also limited data on the optimal management of spindle cell carcinoma. Tumors are commonly large, well-circumscribed, cyst-containing lesions that are most frequently located in the upper outer quadrant of the breast [1]. Biopsy reveals a morphology that consists predominantly of sheets of spindle cells (often >80%), with the tumor exhibiting biological behavior resembling that of a sarcoma. Despite the predominance of spindle cells, Zhu et al. demonstrated that invasive ductal carcinoma (IDC) and ductal carcinoma in-situ (DCIS) components were visualized in 47% of the spindle cell tissue biopsies sampled from a cohort of 19 patients [2]. In congruence with the existing data [3], as well as our present case report, their findings further showed that although immunohistochemical staining in spindle cell carcinoma rarely reveals a hormone receptor-positive disease, a combination of myoepithelial and epithelial markers are frequently observed. These include cytokeratins Pan-K and CK14 (>90% of tissue samples), as well as epithelial membrane antigen (EMA), AE1/3, and CAM 5.2 in 30% to 80% of cases [3-4]. Nuclear positivity for tumor protein p63 is commonly seen.

Although our patient was found to have lymph node metastases on initial presentation, this is an extremely rare presentation for patients with spindle cell carcinoma [5]. Interestingly, such behavior has been found to be associated with tumors that exhibit areas of invasive ductal carcinoma on morphological analysis. This can be confirmed by the presence of invasive ductal components in the affected lymphatic tissue. Extranodal metastases, however, are more frequently observed – commonly to the lungs and bones [3,6]. This is consistent with conventional mammary carcinoma. Although there are no standard treatment guidelines, an extensive analysis of published cases by Moten et al. showed a recent predilection for breast-conserving surgery, with or without radiation [5]. Earlier case reports of spindle cell carcinoma would more commonly feature total mastectomy as the surgical treatment of choice. The evolving approach towards the management of spindle cell carcinoma in the United States is consistent with a global trend towards breast conservation surgery whenever possible. Local excision is important in cases where the lack of targetable hormone receptors limits additional treatment options. The prognosis is usually favorable, especially in early-stage disease, with a cancer-specific 10-year survival rate being >80% in those who undergo a partial mastectomy. Adjuvant radiation, however, has not been found to significantly improve outcomes in those with early- or late-stage disease. Unfortunately, in this particular case, the extent of tumor burden combined with the patient’s poor functional status made chances of survival poor.

Of notable mention is the refractory hypoglycemia that was seen in this case. This has long been documented as a possible complication of malignant neoplasia, yet there are few documented cases of it occurring in spindle cell carcinoma of the breast. Typically of mesenchymal, epithelial, or hematopoietic origin, tumors causing hypoglycemia are classified into 1 of 2 categories: islet cell and non-islet cell tumors. Non-islet cell tumors are thought to decrease blood glucose levels by a combination of factors, such as the secretion of insulin-like growth factor 2 (IGF-II), increased daily glucose utilization, decreased production of growth hormone, and/or metastatic invasion of the liver or adrenal glands, leading to hepatogenic or hypoadrenal hypoglycemia [7]. Unfortunately, in this particular case, serum IGF-II and growth hormone levels were not checked, although at the time it was hypothesized that the persistent hypoglycemia was likely the result of increased glucose utilization secondary to hypermetabolism from the large tumor burden.

We believed it would be of relevance to briefly explore the psychosocial aspect of this case. Had the patient sought treatment when she first discovered the breast mass, the outcome may have been different. Despite advances in early detection of cancers, especially breast malignancies, patients’ denial and reluctance to seek medical care continues to serve as a barrier to effective care. This has long been documented in the literature [8] and continues to be a problem, despite public health efforts to better educate the general population. The etiologies behind patient denial are as diverse as the demographic from which they present. Fear of chronic illness and possible mastectomy, social pressures and responsibilities, cultural beliefs, and past psychiatric history are all considered to play a role. The general public may benefit from learning more about recent improvements in treatment regimens that feature newer drugs and more breast-conserving surgeries, in addition to the current awareness campaigns that tend to focus more heavily on the importance of early detection.

## Conclusions

Spindle cell carcinoma is a rare primary breast malignancy. Tumors are usually large, well-circumscribed, and cystic. In addition to spindle cell morphology, tissues will often display invasive ductal carcinoma and ductal carcinoma in situ components. Lymph node involvement with metastatic disease is uncommon, and the prognosis is often favorable, provided it is diagnosed early and treated promptly. Tumors that are allowed to freely proliferate can advance aggressively with extensive metastases and metabolic derangements. It is imperative that the public is further educated and reassured on the progress being made in the field of breast cancer treatment, as well as our ability as healthcare professionals to compassionately address concerns and fears that may initially serve as barriers to treatment.
